# Bias, Repeatability and Reproducibility of Liver T_1_ Mapping With Variable Flip Angles

**DOI:** 10.1002/jmri.28127

**Published:** 2022-02-27

**Authors:** Sirisha Tadimalla, Daniel J. Wilson, David Shelley, Gavin Bainbridge, Margaret Saysell, Iosif A. Mendichovszky, Martin J. Graves, J. Ashley Guthrie, John C. Waterton, Geoffrey J.M. Parker, Steven P. Sourbron

**Affiliations:** ^1^ Institute of Medical Physics University of Sydney Sydney Australia; ^2^ Department of Biomedical Imaging Sciences University of Leeds Leeds UK; ^3^ Leeds Teaching Hospitals Trust Leeds UK; ^4^ Department of Radiology University of Cambridge Cambridge UK; ^5^ Bioxydyn Ltd Manchester UK; ^6^ Centre for Imaging Sciences, Division of Informatics Imaging and Data Sciences, School of Health Sciences, Faculty of Biology Medicine and Health, University of Manchester Manchester Academic Health Sciences Centre Manchester UK; ^7^ Centre for Medical Image Computing, Department of Medical Physics and Biomedical Engineering University College London London UK; ^8^ Department of Infection, Immunity and Cardiovascular Disease University of Sheffield Sheffield UK

**Keywords:** T_1_ mapping, liver, VFA, reproducibility, repeatability, bias, precision

## Abstract

**Background:**

Three‐dimensional variable flip angle (VFA) methods are commonly used for T_1_ mapping of the liver, but there is no data on the accuracy, repeatability, and reproducibility of this technique in this organ in a multivendor setting.

**Purpose:**

To measure bias, repeatability, and reproducibility of VFA T_1_ mapping in the liver.

**Study Type:**

Prospective observational.

**Population:**

Eight healthy volunteers, four women, with no known liver disease.

**Field Strength/Sequence:**

1.5‐T and 3.0‐T; three‐dimensional steady‐state spoiled gradient echo with VFAs; Look‐Locker.

**Assessment:**

Traveling volunteers were scanned twice each (30 minutes to 3 months apart) on six MRI scanners from three vendors (GE Healthcare, Philips Medical Systems, and Siemens Healthineers) at two field strengths. The maximum period between the first and last scans among all volunteers was 9 months. Volunteers were instructed to abstain from alcohol intake for at least 72 hours prior to each scan and avoid high cholesterol foods on the day of the scan.

**Statistical Tests:**

Repeated measures ANOVA, Student *t*‐test, Levene's test of variances, and 95% significance level. The percent error relative to literature liver T_1_ in healthy volunteers was used to assess bias. The relative error (RE) due to intrascanner and interscanner variation in T_1_ measurements was used to assess repeatability and reproducibility.

**Results:**

The 95% confidence interval (CI) on the mean bias and mean repeatability RE of VFA T_1_ in the healthy liver was 34 ± 6% and 10 ± 3%, respectively. The 95% CI on the mean reproducibility RE at 1.5 T and 3.0 T was 29 ± 7% and 25 ± 4%, respectively.

**Data Conclusion:**

Bias, repeatability, and reproducibility of VFA T_1_ mapping in the liver in a multivendor setting are similar to those reported for breast, prostate, and brain.

**Level of Evidence:**

1

**Technical Efficacy Stage:**

1

Renewed interest in T_1_ as a quantitative imaging biomarker (QIB) has sparked an increase in research and development of fast and accurate T_1_ mapping methods across the body.^1^, ^2^, ^3^, ^4^ A wide range of traditional methods for T_1_ mapping are available, which include fully relaxed methods such as inversion‐recovery (IR), Look‐Locker (LL), and steady‐state methods such as variable flip angle (VFA) or variable repetition time saturation recovery (VTR). IR methods are the most accurate^5^ but are usually too slow for practical use, and VTR methods are not routinely used in clinical settings. LL type methods including modified LL imaging (MOLLI) are commonly used in abdomen and thorax for multislice, two‐dimensional T_1_ mapping.^6^ These methods are valued for their high reproducibility^7^ but are unsuitable for applications that require volumetric coverage, as in liver disease. In such applications, there is a rationale for VFA methods, which allow fast, three‐dimensional T_1_ mapping of large volumes. VFA is widely used, and the past few years in particular have seen an increase in the application of VFA techniques in organs such as brain, breast, and prostate.^8^, ^9^, ^10^

A downside of VFA methods is that they are more susceptible to bias caused by $B_{1}^{+}$ nonuniformities, imperfect spoiling, and magnetization transfer (MT) effects.^5^, ^11^ These errors depend on scanner hardware and sequence optimization and may vary spatially across the field of view, affecting accuracy, intrascanner repeatability, and interscanner reproducibility in multicenter clinical trials or diagnostic methods that require relaxation time measurements, for example, for the assessment of treatment response.

A multicenter phantom study across 10 scanners of three vendors and two field strengths using VFA T_1_ mapping found that the combined effect of these errors can be substantial, producing a bias up to 32%, intrascanner relative error (RE) (Different definitions of repeatability and reproducibility metrics are in common use. Due to repeatability effects up to 26% and interscanner reproducibility RE of 22% at 1.5 T and 45% at 3.0 T.^12^ (Different definitions of repeatability and reproducibility metrics are in common use. For the purposes of this paper, whenever literature values are cited, they are converted to the RE definition used in this study [see Methods section] to allow direct numerical comparison between results of this study and the literature.) While such phantom studies are a valuable and necessary contribution to characterizing the performance of quantitative measurements, their findings cannot be used directly to infer performance in vivo, due to subject‐ and organ‐specific sources of variation. These include $\begin{array}{l} B_{1}^{+} \end{array}$ errors caused by nonuniform RF penetration and standing wave effects, and the impact of physiological motion including breathing and blood flow on the measurements.

Correction techniques for $\begin{array}{l} B_{1}^{+} \end{array}$ errors have been proposed,^13^, ^14^ and multisite studies in organs such as the brain^15^ and breast^16^, ^17^ suggest this improves repeatability and reproducibility. In the brain, a multiparametric VFA protocol with corrections for $\begin{array}{l} B_{1}^{+} \end{array}$ and imperfect spoiling on six 3.0 T scanners from two vendors reported repeatability‐ and reproducibility RE for *R*_1_ (=1/T_1_) up to 16% and 20%, respectively.^8^ In breast fibroglandular tissue, a single vendor study reported reproducibility RE across three sites of 14% in VFA T_1_ after $\begin{array}{l} B_{1}^{+} \end{array}$ correction at 3.0 T.^9^ Similar values were found in the prostate.^10^ However, these studies do not provide a comprehensive coverage of the two main clinical field strengths and three main vendors. As a result, biases, repeatability, and reproducibility RE may be underestimated.

In the liver, VFA T_1_ mapping has been proposed to assess conditions such as liver fibrosis and cirrhosis and for calibration of dynamic contrast enhanced (DCE) MRI.^18^, ^19^, ^20^, ^21^, ^22^, ^23^, ^24^, ^25^, ^26^, ^27^ A few studies of repeatability and/or reproducibility of liver T_1_ have been reported but to our knowledge none employed VFA and therefore do not address the influence of varying $\begin{array}{l} B_{1}^{+} \end{array}$ fields on VFA‐derived liver T_1._ ^28^, ^29^ It is not guaranteed that results in relatively static organs like the brain, breast, and prostate will translate to the liver, which exhibits significant deformable breathing motion and may be more susceptible to inhomogeneities due to its large size.

Thus, the aim of this study was therefore to determine the bias, repeatability, and reproducibility of VFA T_1_ mapping in the liver, in real‐world conditions at 1.5 T and 3.0 T and in scanners of three main vendors in widespread use today. These values might be used in uncertainty analysis and for estimating study power and will establish a baseline against which subsequent methodological developments can be benchmarked.

## Materials and Methods

### Subjects

Eight healthy volunteers (age = 23–58 years, mean 37 years; 4 women) with no known liver disease or MRI contraindications were scanned twice each (between 30 minutes to 3 months apart) on six MRI scanners. The maximum period between the first and last scans among all volunteers was 9 months. The study was approved by the institutional research ethics committee (University of Leeds, Faculty of Medicine and Health: MREC17‐111), and written informed consent was obtained from all volunteers. Volunteers were instructed to abstain from alcohol intake for at least 72 hours prior to each scan and to avoid high cholesterol foods on the day of the scan.

### Scanners

Details of the scanners and coils used are summarized in Table 1. A total of six scanners (two field strengths × three vendors [GE Healthcare, Philips Medical Systems, and Siemens Healthineers]) located across four different sites were used.

**TABLE 1:** Details of Scanners Used in This Study

Scanner	Vendor	Model	RF Coil
S1	Siemens Healthineers	3.0 T Prisma VE11C	18 channel array
S2	Siemens Healthineers	1.5 T Aera VE11A	18 channel array
P1	Philips Medical Systems	3.0 T Achieva	32 channel array
P2	Philips Medical Systems	1.5 T Ingenia	dStream Torso
G1	GE Healthcare	3.0 T Discovery MR750	32 channel array
G2	GE Healthcare	1.5 T Discovery MR450	32 channel array

### MRI Protocol

The MRI protocol was developed initially on the Siemens 3.0 T scanner and then transferred as closely as possible on the other scanners. Where an exact one‐to‐one correspondence of sequence parameters was not possible, the spatiotemporal geometries were aligned first (acquired voxel size, field of view (FOV), and acquisition time) to ensure a fair comparison of scanners in terms of signal to noise ratio (SNR) and to match breath‐hold times, after which contrast and other parameters were optimized to match the reference protocol as closely as possible. Pilot data were acquired on standardized phantoms and volunteers to ensure sufficient image quality.

The protocol consisted of standard localizer/survey and calibration scans, followed by multislice two‐dimensional anatomical reference T_2_‐weighted scans in the 1) coronal and 2) transverse planes with full liver coverage, 3) where available, a reference two‐dimensional T_1_ mapping sequence using an LL or MOLLI type sequence, 4) a three‐dimensional coronal RF spoiled gradient echo (SPGR) breath‐hold (BH, ~16 seconds) sequence with six flip angles (VFA BH), and 5) a fast (~2 seconds) three‐dimensional coronal free‐breathing (FB) RF spoiled SPGR sequence with six flip angles (VFA FB). The fast sequence was acquired continuously for up to a minute to average out breathing motion.

Two‐dimensional LL T_1_ mapping was implemented as a reference on the Siemens 3.0 T scanner using a nonselective IR magnetization preparation and gradient echo readout, with a simulated heart rate of 80 beats/minute to ensure sufficient sampling of the recovery curve for liver T_1_. On the Philips 1.5 T and 3.0 T scanners, a dedicated MOLLI sequence was used with simulated electrocargiogram (ECG) using the same heart rate of 80 beats/minute. The two‐dimensional MOLLI sequence was not available on the other three scanners.

Three‐dimensional VFA T_1_ mapping was implemented on the Siemens scanners using a three‐dimensional Fast Low Angle Shot (FLASH) sequence and on the Philips scanners using a T_1_‐fast field echo sequence. Three‐dimensional T_1_‐weighted images were acquired with a set of six flip angles, with receiver gains set using a preparation scan at 15°. Preparation scans were turned off for subsequent flip angles to ensure a constant receiver gain. On the GE scanners, it was not possible to automatically (without user‐controlled manual prescan) prevent a change of the receiver gain between different flip angle acquisitions using the product three‐dimensional Fast Spoiled Gradient Echo (FSPGR) sequence. It was also not possible to set up the VFA FB sequence with multiple measurements for each flip angle acquisition. Severe phase‐wrap artifacts were also observed when using the FSPGR product sequence for coronal VFA acquisition. Therefore, a modified version of the FSPGR sequence, named the multiphase multiflip angle (MPMFA) sequence, was developed in‐house. The MPMFA sequence allowed 1) single acquisition for all flip angles with a fixed receiver gain set at flip angle 15°, 2) multiple measurements within acquisition of each flip angle, and 3) a rotated slab excitation to reduce phase‐wrap artifacts and sufficient anterior posterior coverage. The code for the MPMFA sequence will be made available upon request from sites with access to the GE research sharing database. $\begin{array}{l} B_{1}^{+} \end{array}$ mapping was not implemented on any scanner due to lack of product mapping sequences on all scanners.

Detailed imaging parameters for each scanner are given in Table S1.

### Image Processing

Anonymized images were transferred from all scanners in DICOM format. Image processing was performed centrally by a single user (S.T.—9 years of experience) using the open‐source software PMI (https://github.com/plaresmedima/PMI-0.4) customized for this purpose (compiled version freely available as supplementary material at https://doi.org/10.5281/zenodo.5589509). MOLLI T_1_ maps were obtained by fitting to signal intensities as a function of inversion time as described by Messroghli et al.^30^ VFA T_1_ maps were obtained by fitting signal intensities at the six flip angles with the linearized steady‐state SPGR equation.^5^ Continuously acquired free‐breathing three‐dimensional coronal SPGR images were motion‐corrected by using nonrigid registration with free form deformation between each image and magnitude averaged prior to VFA T_1_ mapping.^31^ As the study was performed on healthy volunteers with no known liver disease, liver fat and iron levels were assumed to be normal and no corrections were applied during T_1_ calculations. Liver regions of interest (ROIs) were drawn on the T_1_ maps as follows: 1) a central slice containing the portal vein at its largest was chosen; and 2) the entire liver within the slice was manually outlined and large blood vessels were removed from the ROI by user‐defined thresholding. The user was not blinded to time points or subjects and compared segmentations from the same subject across scans to avoid intrareader segmentation differences impacting on the result.

### Statistical Analysis

Median T_1_ values within the ROIs were extracted from each T_1_ map. Bias estimate, repeatability, reproducibility, and spatial inhomogeneity were calculated for each volunteer as described below, and averages over the volunteers were reported along with their standard error.

$$\begin{array}{l} \text{Bias estimate}\left(\%\right)=100\times \begin{array}{l} \frac{T_{1}\left(\text{averaged over scans}\;1\;\text{and}\;2\right)—\text{reference}\;T_{1}}{\text{reference}\;T_{1}} \end{array} \end{array}$$

$$\text{Repeatability}\;\mathrm{RE}\left(\%\right)=100\times 1.96\times \frac{\text{Standard deviation}\;\left(\text{scan}1,\text{scan}2\right)}{\text{Average}\;\left(\text{scan}1,\text{scan}2\right)}$$

$$\text{Reproducibility}\;\mathrm{RE}\;\left(\%\right)=100\times 1.96\times \frac{\text{Standard deviation}\;\left(\mathrm{all}\;\text{vendors}\right)}{\text{Average}\;\left(\mathrm{all}\;\text{vendors}\right)}$$

$$\begin{array}{l} \text{Spatial heterogeneity}\left(\%\right)=100\times \frac{\text{Inter quartile range}\;T_{1}}{\text{Median}\;T_{1}},\text{averaged over scans}\;1\;\text{and}\;2 \end{array}$$

The repeatability RE measures the relative random error (half of the 95% confidence interval, CI) on median T_1_ when all measurements are done on the same machine; the reproducibility RE measures the relative random error (half of the 95% CI) on median T_1_ when measurements are performed on machines from different vendors. Reference T_1_ values in the liver were obtained from literature for healthy liver (752 msec at 3.0 T, 602 msec at 1.5 T).^32^ Separate pairwise reproducibility REs were also calculated for the three possible pairs of vendors.

Between‐subject variation in T_1_, estimated for each scanner and method, was calculated as,

$$\text{Between}\text{subject variation}\;\left(\%\right)=100\times 1.96\times \frac{\text{Standard deviation}\;\left(\mathrm{all}\;\text{subjects}\right)}{\text{Average}\;\left(\mathrm{all}\;\text{subjects}\right)}$$

Comparisons of means across subjects, sequences, and scanners were performed using repeated measures analysis of variance (rANOVA). When the *P*‐value was less than 0.05, post hoc pairwise *t*‐tests were performed. Levene's test was used to compare between‐subject measurement variances across sequences and scanners.

### Incidental Findings

Scans were also read by a radiologist so that any incidental findings could be followed up confidentially: any such findings were not disclosed to the investigators and so are not reported in this paper.

## Results

All volunteers completed the study except one who withdrew after completing scans on three of the six scanners. All acquired data were included in the analysis.

### Summary Parameters

Table 2 presents the 95% CI on the overall bias estimate, repeatability, reproducibility, and spatial heterogeneity of MOLLI and VFA T_1_ values in the liver. The table shows that VFA overestimates median liver T_1_ by 30% on average, relative to literature estimates. The RE is ±10% if all scans are performed on a single scanner and ±27% if scanners from different vendors and field strengths are used. Bias estimate, repeatability, and reproducibility of MOLLI T_1_ measurements are consistent with those reported in the literature using IR/LL T_1_ mapping methods in the liver.^29^, ^32^ More detailed performance metrics for each sequence, field strength, and vendor are provided in Table S2. When compared with MOLLI, VFA T_1_ values are 15 times more biased, four times less repeatable, two times less reproducible, and two times less homogeneous. Table 3 compares the repeatability and reproducibility in liver T_1_ as measured in this study against other results in breast, brain, and prostate after correcting for different definitions of repeatability and reproducibility. Corrections applied to convert literature definitions of repeatability and reproducibility to the definitions used in this study are given in Table S3.

**TABLE 2:** The 95% CI on Overall Mean Bias, Repeatability, Reproducibility, and Spatial Heterogeneity of T_1_ Values in the Normal Liver

	MOLLI	VFA BH	VFA FB
Bias (%)	1.7 ± 3.7**	31 ± 8.0	29 ± 8.1
Relative repeatability Error (%)	2.4 ± 0.7**	11 ± 3.1	9.4 ± 2.0
Relative reproducibility error (%) at 1.5 T		34 ± 9.2^a^	24 ± 8.1
Relative reproducibility error (%) at 3.0 T	14 ± 5.8*	22 ± 4.0^a^	29 ± 7.1
Spatial heterogeneity (%)	11 ± 1.5**	25 ± 1.9^a^	18 ± 2.3

MOLLI, modified Look‐Locker imaging; rANOVA, repeated measures ANOVA; VFA, variable flip angle.

^a^ Paired *t*‐test (VFA BH vs. VFA FB) *P*‐value < 0.0001.

**rANOVA *P*‐value < 0.0001; *rANOVA *P*‐value < 0.01.

**TABLE 3:** Repeatability and Reproducibility of VFA T_1_ Values in the Liver, Compared to Literature Values in Phantoms and Other Organs

Application Area	Corrections	Scanners	Relative Repeatability Error^a^ (%)	Relative Reproducibility Error^a^ (%)
Liver (from this study)	None	1.5 T: 1 × S, 1 × P, 1 × G 3.0 T: 1 × G, 1 × P, 1 × S	10%	28%
Breast (reference 8)	$\begin{array}{l} B_{1}^{+} \end{array}$ correction	3.0 T: 3 × S	11%	14%
Brain (R_1_) (reference 7)	$\begin{array}{l} B_{1}^{+} \end{array}$ correction; imperfect spoiling	3.0 T: 4 × S, 2 × P	10%–16%	10%–20%
Prostate (transitional zone) (reference 9)	With and without $\begin{array}{l} B_{1}^{+} \end{array}$ correction	3.0 T: 2 × S	12% (14% without correction)	14% (18% without correction)

VFA, variable flip angle.

^a^ Literature values were converted to the definition used in this study.

### Effect of Field Strength

**FIGURE 1:**
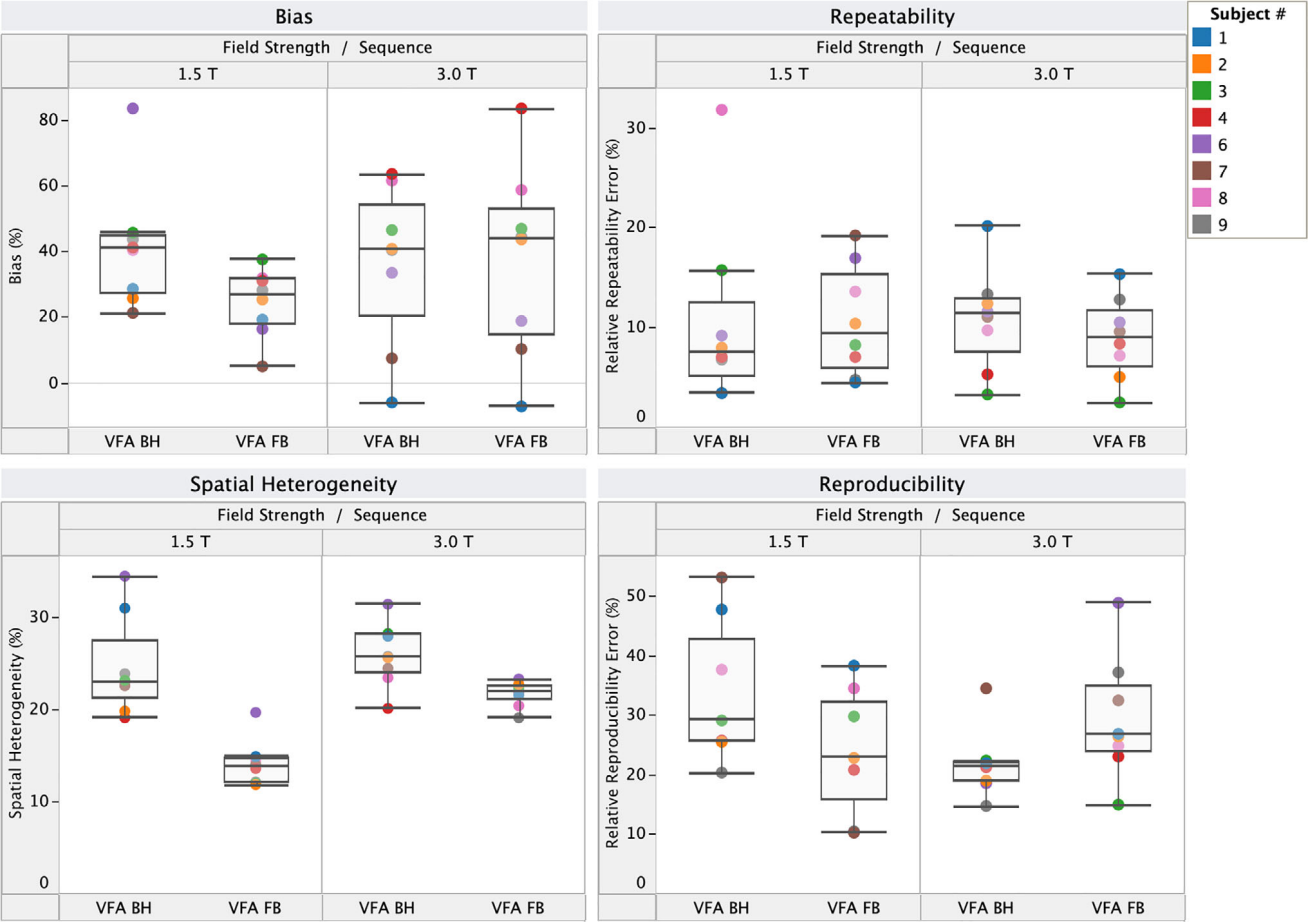
Bias, repeatability, reproducibility, and spatial heterogeneity of variable flip angle (VFA) T_1_ measurements across field strengths. The box plots indicate the median and interquartile range (IQR), and the whiskers encompass all points within 1.5 times the IQR. Each color‐coded datapoint represents a single volunteer.

Figure 1 shows the bias, repeatability, reproducibility, and spatial heterogeneity of VFA T_1_ measurements for the two clinical field strengths separately. Results of the corresponding rANOVA and Levine's tests are given in Table 4. Unlike the VFA FB sequence, the T_1_ bias was not significantly different between field strengths for the VFA BH sequence, despite increased $\begin{array}{l} B_{1}^{+} \end{array}$ nonuniformity expected at 3.0 T. However, the variance in the T_1_ bias was larger at 3.0 T for the VFA FB sequence, in line with a higher spatial heterogeneity for this sequence at 3.0 T. While field strength had no effect on the repeatability, both the mean and the variance of the relative reproducibility error were lower at 3.0 T for VFA BH.

**TABLE 4:** Results (*P*‐values) of Repeated Measures ANOVA (Levene's Tests in Parentheses) on the Effect of Field Strength on Bias, Repeatability, Reproducibility, and Spatial Heterogeneity

Sequence	Bias	Repeatability	Reproducibility	Spatial Heterogeneity
MOLLI	**<0.001** (0.70)	0.29 (0.36)	‐	**<0.0001** (0.30)
VFA BH	0.16 (0.30)	0.99 (0.77)	**0.01 (0.03)**	0.30 (0.26)
VFA FB	**0.004 (0.006)**	0.69 (0.75)	0.68 (0.17)	**<0.0001** (0.31)

Significant results are highlighted in bold font.

MOLLI, modified Look‐Locker imaging; VFA, variable flip angle.

### Effect of Vendor

**FIGURE 2:**
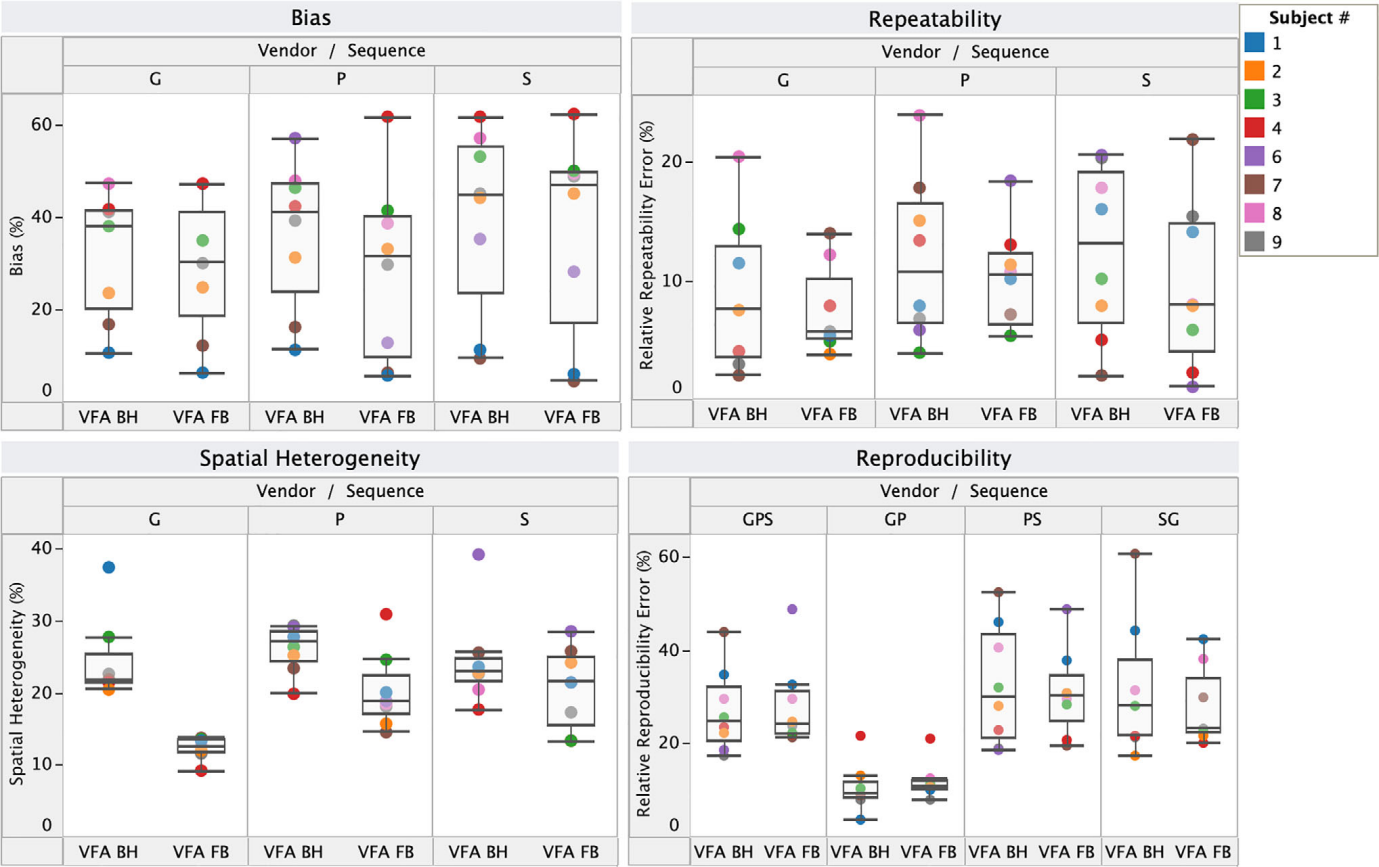
Bias, repeatability, reproducibility, and spatial heterogeneity of variable flip angle (VFA) T_1_ measurements across vendors. The box plots indicate the median and interquartile range (IQR), and the whiskers encompass all points within 1.5 times the IQR. Each color‐coded datapoint represents a single volunteer.

Figure 2 summarizes the bias, repeatability, reproducibility, and spatial heterogeneity of VFA T_1_ measurements across the three vendors (S, P, and G), and for the possible pairs of two vendors for both field strengths. Results of the corresponding rANOVA and Levene's tests are given in Table 5. Vendor has no effect on repeatability of liver VFA T_1_ values; however, variance in bias on vendor S scanners is significantly higher than on vendors G and P. This manifests also as a significant improvement in reproducibility when vendor S is removed. Vendor choice also affects spatial heterogeneity, as seen by the lower mean and variance in heterogeneity in VFA FB T_1_ values on vendor G.

**TABLE 5:** Results (*P*‐Values) of Repeated Measures ANOVA (Levene's Tests in Parentheses) on the Effect of Vendor on Bias, Repeatability, and Spatial Heterogeneity

Sequence	Bias	Repeatability	Reproducibility	Spatial Heterogeneity
MOLLI^a^	**0.003** (0.30)	0.96 (0.88)	**‐**	**0.002** (0.58)
VFA BH	0.08 (**0.005**)	0.69 (0.80)	**<0.0001** (0.05)	0.31 (0.65)
VFA FB	0.69 (**0.0003**)	0.40 (0.13)	**<0.0001** (0.22)	**0.007 (0.003)**

Significant results are highlighted in bold font.

MOLLI, modified LL imaging; VFA, variable flip angle.

^a^ Vendors P and S only.

### Effect of Sequence Optimization

No significant differences in bias or repeatability were found between the VFA BH and VFA FB methods (*P* = 0.22 and 0.29, respectively). However, reproducibility errors were significantly different at both the field strengths. The use of very low spatial resolution during imaging with VFA FB resulted in significantly lower spatial heterogeneity than the BH approach.

### Comparison of T_1_ Values

**FIGURE 3:**
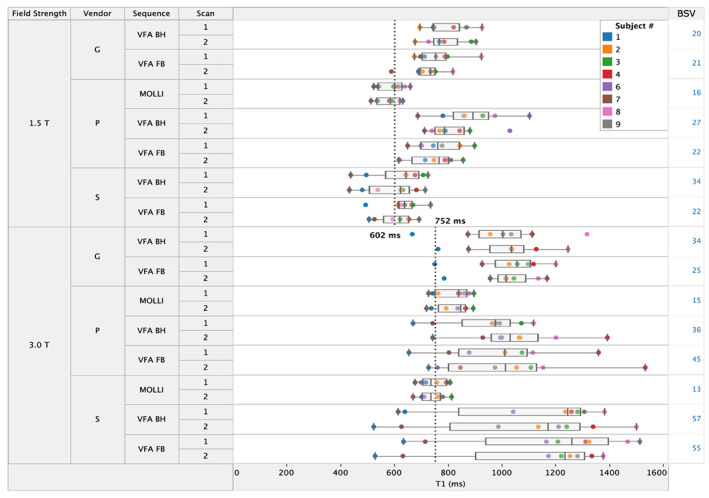
T_1_ values in the normal liver at 1.5 T and 3.0 T. Each color‐coded datapoint represents a single volunteer. Between‐subject variation (BSV, expressed as a percentage) is given for each scanner and method on the right.

T_1_ values in the normal liver in all volunteers on all six scanners are provided as supplementary material (https://doi.org/10.5281/zenodo.5589509) and presented in Fig. 3. The 95% CI of the mean T_1_ values at 1.5 T and 3.0 T are summarized in Table 6. The data confirm that in general the average VFA T_1_ is overestimated relative to the MOLLI reference (as seen from the bias estimates). One notable exception is the VFA T_1_ measurements on vendor S at 1.5 T which are close to the corresponding MOLLI T_1_ measurements.

**TABLE 6:** The 95% CI of the mean T_1_ values in the liver at 1.5 T and 3.0 T

	Literature reference	MOLLI	VFA BH	VFA FB
T_1_ at 1.5 T (msec)	602	586 ± 33	780 ± 91	705 ± 39
T_1_ at 3.0 T (msec)	752	782 ± 36	1036 ± 148	1053 ± 147

MOLLI, modified Look‐Locker imaging; VFA, variable flip angle.

### Between‐Subject Variation

Between‐subject variation measured in T_1_ values within the population of volunteers in this study for each scanner and method are shown in Fig. 3. Overall, between‐subject differences in measured T_1_ values are significant for both MOLLI and VFA methods. The MOLLI method estimates a between‐subject variation of approximately 15%, irrespective of vendor and field strength. With the VFA methods, subject‐wise differences are larger, the degree depending on both field strength as well as vendor. The scanner from vendor S at 3.0 T generates the largest between‐subject variation with both VFA methods, at 56% on average.

## Discussion

In this work, bias, repeatability, reproducibility, and spatial heterogeneity of VFA T_1_ values were measured in the liver of healthy volunteers, on a representative set of six scanners at two field strengths from three vendors.

Bias in VFA T_1_ values are rarely reported in vivo, but the values reported in this study were smaller than those reported in a standardized phantom.^12^, ^33^ Bias was calculated relative to reference liver T_1_ measurements at the two field strengths obtained from literature; the literature values used as reference were close to the MOLLI T_1_ measurements. Root‐mean‐squared deviation of MOLLI, VFA BH, and VFA FB T_1_ measurements in this study from literature values are 3.4%, 25%, and 23%, respectively.^32^

The between‐subject variation in liver T_1_ estimated by the MOLLI method is also consistent with the literature, while the variation in VFA T_1_ values between subjects is considerably larger.^32^ This is consistent with the effect of known errors in VFA‐based T_1_ measurement, in particular the impact of B_1_ effects, which are known to cause nonuniform excitations across the liver in a manner that depends on body size. The hypothesis is also supported by the observed trends in the spatial heterogeneity measurements. At 1.5 T, the VFA FB shows a comparable spatial heterogeneity to the MOLLI, consistent with the observation that the between‐subject variability at 1.5 T is similar between MOLLI and VFA FB. At 3.0 T, the spatial heterogeneity of the VFA FB is substantially larger than MOLLI for vendors P and S, but not for vendor G, again in agreement with the observed T_1_‐variability in the population. The data also indicate that these errors are to some extent reproducible. For example, low liver T_1_ values are recorded for subjects 1 and 7, and high liver T_1_ values are recorded for subject 4 consistently across all scanners and methods.

Repeatability and reproducibility of VFA T_1_ in the liver as measured in this study are comparable to other static organs.^8^, ^9^, ^10^ The repeatability RE in liver (10% ± 2%) is in fact at the lower end of published results in brain, breast, and prostate (10%–16%). The reproducibility RE in the liver (29% ± 7% at 1.5 T and 25% ± 4% at 3.0 T) is higher than previous studies (7%–20% at 3.0 T). These comparisons should be interpreted with caution as published studies are positively biased, using a narrower range of scanners (one field strength and no more than two vendors). Indeed, restricting the liver T_1_ reproducibility to pairs of vendors improves the reproducibility RE to 10%–12% for the best aligned pair of vendors (G–P), well within the range of previous studies in other organs.

Unlike the repeatability and reproducibility studies on VFA T_1_ mapping in the other organs, liver VFA T_1_ values obtained in this study were acquired without corrections for $\begin{array}{l} B_{1}^{+} \end{array}$ effects, imperfect spoiling, MT effects, or other confounders. Correcting for these effects may improve the reproducibility, but evidence for this is limited, especially in view of the indication above that B_1_ errors themselves may be reproducible. While previous multivendor studies in brain, breast, and prostate with $\begin{array}{l} B_{1}^{+} \end{array}$ corrections showed improved reproducibility relative to an earlier phantom study, these studies also used a narrower sample of vendors, scanners, or field strengths. This potentially produced an optimistic assessment of repeatability and reproducibility. Only one multivendor study, in the prostate, assessed the impact of $\begin{array}{l} B_{1}^{+} \end{array}$ correction directly and found the improvement to be modest, improving the reproducibility RE from 18% to 14%.^10^ This is consistent with our observation that results in the absence of $\begin{array}{l} B_{1}^{+} \end{array}$ correction are in the range of $\begin{array}{l} B_{1}^{+} \end{array}$ corrected results in other organs. Hence, the room for improvement in reproducibility using standard $\begin{array}{l} B_{1}^{+} \end{array}$ correction methods may be limited. However, the data in this study suggest that $\begin{array}{l} B_{1}^{+} \end{array}$ correction may have a significant impact on the overall bias and accuracy of the measurements on single‐subject level. In the liver, $\begin{array}{l} B_{1}^{+} \end{array}$ inhomogeneities of 0.4–1.3 (ratio of actual to prescribed flip angle) have been reported at 3.0 T.^34^ For the literature, T_1_ value of 752 msec at 3.0 T, assuming a TR = 3.5 msec, bias in VFA T_1_ measurements between −84% and 70% can be expected, which are much larger than the bias estimates obtained in this study.

A separate issue is that fast and validated $\begin{array}{l} B_{1}^{+} \end{array}$ correction methods are not routinely available on all clinical scanners and are therefore of limited use in clinical trials or clinical practice today. Indeed, in its recent profile revision, the QIBA DCE‐MRI Biomarker Committee has specifically not included $\begin{array}{l} B_{1}^{+} \end{array}$ mapping as a requirement for VFA T_1_ mapping due to “the dearth of literature and lack of access to vendor‐specific $\begin{array}{l} B_{1}^{+} \end{array}$ mapping sequences” (QIBA DCE‐MRI BC Call Summary, 21 Dec 2020, [http://qibawiki.rsna.org/images/d/d7/2020_12-21_QIBA_DCE-MRI_BC_Call_Summary-FINAL.pdf]). The committee also highlighted the lack of test–retest data on the effects of $\begin{array}{l} B_{1}^{+} \end{array}$ corrections on T_1_ measurements in routine VFA T_1_ mapping. This is crucial because $\begin{array}{l} B_{1}^{+} \end{array}$ corrections may themselves be subject to measurement error.^35^ Indeed, there have been reports of exacerbation of $\begin{array}{l} B_{1}^{+} \end{array}$ nonuniformity in some vendor‐provided maps^36^ and increase in bias in T_1_ values after inline $\begin{array}{l} B_{1}^{+} \end{array}$ corrections.^37^ These observations indicate the importance of robust and accurate $\begin{array}{l} B_{1}^{+} \end{array}$ correction, which may come at a substantial cost in acquisition time.

Comparison with MOLLI indicates that there is significant room to improve on the accuracy of VFA and supports the common assumption that the faster acquisition afforded by VFA comes at a cost of accuracy, repeatability, and reproducibility. It may be likely that the differences between VFA and MOLLI as reported in this study are overestimated; as the MOLLI sequence was only available on three of the six scanners, its reproducibility is likely to be lower in a more representative population of scanners. However, repeatability of the MOLLI T_1_ in this study is very consistent with literature using LL methods, while the repeatability RE of liver VFA T_1_ is substantially higher.^32^

On the effect of field strength, the only significant differences between 1.5 T and 3.0 T are an improved reproducibility at 3.0 T for the BH VFA sequence, but a larger between‐subject variability and spatial heterogeneity for the VFA FB. Hence, it appears the optimal field strength in terms of reproducibility is sequence specific, with 3.0 T preferred for the BH sequence and 1.5 T preferred for the FB sequence. Considering the results of individual vendors separately indicates that the optimal field strength is also vendor specific. In all three vendors, the between‐subject variation increased at 3.0 T in line with subject‐specific errors caused by B1‐effects. In vendor S, the mean bias is larger at 3.0 T, whereas for vendors P and G it is comparable. On the whole, this indicates a preference for 1.5 T when using uncorrected VFA in view of the smaller between‐subject variability and spatial heterogeneity.

On the effect of vendor, including vendor S in a study increases the reproducibility RE substantially relative to studies that include vendors G and P only. This observation remains valid when field strengths are considered separately. Between the other vendors G and P, results are comparable, the only distinguishing feature being a lower spatial heterogeneity for vendor G at 3.0 T for the VFA FB. On the other hand, dedicated sequence development was needed to enable this study on vendor G, unlike the other vendors where product sequences were available. A different picture emerges when considering the field strengths separately. Unlike at 3.0 T, vendor S has the lowest bias at 1.5 T, showing a systematic error that is substantially smaller than vendors G and P. The repeatability RE for vendor S at 1.5 T is also lower than that of vendors G and P, though the differences are smaller. This indicates that vendor S reduces the reproducibility at 1.5 T only because it has a substantially lower bias—illustrating the limitation of using reproducibility measures alone to characterize an imaging biomarker assay.^38^

To test the effect of sequence optimization on the performance of VFA T_1_ mapping, we included two sequences in the study that were optimized in different ways: a BH sequence at high spatial and low temporal resolution, and an FB sequence at low spatial and high temporal resolution. Reproducibility RE of the FB sequence was lower at 1.5 T and higher at 3.0 T compared to the BH sequence. And other than an improved spatial heterogeneity of the FB sequence, all remaining parameters were comparable between the sequences, indicating that the details of sequence optimization do not fundamentally impact on the accuracy of the measurement. Hence, the choice of sequence settings can be based on other criteria, such as the need for high spatial detail or a desire to avoid breath holds in frail patient populations.

### Study Limitations

Only one scanner of each vendor and each field strength was available for this multivendor study, and therefore, we are not able to test the effect of the variability induced by using two different scanners of the same make and model. Naturally, the small sample size of eight healthy volunteers is a limitation and has reduced the study's power to detect more subtle differences in means. Finally, bias in VFA T_1_ in the liver could not be determined due to the lack of a true reference measurement in the study. Hence, we provided an estimated bias using a literature value. This was close to the measured MOLLI values in our population, which provides some confidence that the literature value is close to the ground truth.

Liver T_1_ measurements are known to be affected by fat, iron, and glycogen content.^39^, ^40^ In this healthy volunteer study, liver fat and iron levels were assumed to be normal. While this may cause bias in subjects where these assumptions are invalid, both fat and iron levels can be assumed to remain stable throughout the study period. Their effect on T_1_ repeatability and reproducibility should, therefore, be minimal, which was the main focus of this study. Participants in this study were also not instructed to attend scans in consistent fasted or fed states, and variation in liver glycogen levels between scans can impact T_1_ repeatability and reproducibility. However, variations in T_1_ in healthy volunteers between fed and fasted states^40^ have been reported to be within the same‐day test–retest T_1_ repeatability ranges reported in volunteers in a fasting state.^29^ Therefore, the impact of variation in meal intake between scans is not expected to have a major impact on the repeatability and reproducibility REs obtained in this study.

Another limitation of this study is that the optimization of sequences on each scanner was not independent. Some sequence parameters such as FOV, spatial resolution, and FA were kept fixed; however, it was not possible to directly match parameters such as parallel imaging acceleration factors or phase oversampling across scanners. For example, differences in acquisition parameters such as TR are known to affect the sensitivity of VFA T_1_ measurements. For a given T_1_, the Ernst angle increases with increasing TR.^41^ However, the range of TR values used in this study was 3.19–6.04 msec. For the literature values of liver T_1_, the range of e−TRT1 is 0.99–0.996. The corresponding shift in the Ernst angle is 3°; therefore, the impact of the mismatch in TR to VFA T_1_ reproducibility RE is expected to be negligible. While an effort was made to minimize differences in sequence implementation on the scanners, any remaining differences could have contributed to the vendor and field strength effects.

In this study, the MOLLI measurements were performed in the transverse orientation, while the VFA T_1_ images were acquired coronally. VFA T_1_ mapping in the liver is often used for the calibration of DCE‐MRI signals. In such a liver MRI protocol, the VFA acquisition is required to match the DCE‐MRI acquisition. While the current clinical norm is to acquire liver DCE‐MRI in the transverse orientation, coronal acquisitions are preferred to avoid inflow effects in arterial input function measurements for pharmacokinetic modeling and to simplify motion correction. Therefore, in this study, the VFA T_1_ acquisitions were performed coronally. On the other hand, the MOLLI is performed transverse as a standard for liver T_1_ studies. In retrospect, coronal MOLLI acquisition could have allowed direct comparisons with VFA. However, no differences in renal T_1_ values between coronal and axial acquisitions were found in other studies.^42^ Therefore, the impact of different acquisition orientations of VFA and MOLLI T_1_ comparisons is not expected to be large.

On vendor G, the sequence was additionally modified at a sequence programming level in order to enable scans of all flip angles to run consecutively, without phase‐wrap artifacts in the coronal acquisition with a large field of view, and no change in receiver gain between flip angles, whereas in vendors S and P only the sequence parameters were optimized. This may also have created a bias in favor of vendor G. Finally, the sequences were first set up and tested on a single vendor and a single field strength and subsequently translated to others. It is plausible that this has created a bias in favor of the reference scanner (vendor S, 3.0 T).

### Conclusion

Bias, repeatability, and reproducibility of VFA T_1_ mapping in the liver in a multivendor setting are similar to those reported in breast, prostate, and brain. The numerical values reported in this study can serve as benchmarks against which any future improvements of VFA T_1_ mapping in the liver can be qualified.

## Supporting information


**Table S1** Imaging protocol and optimized sequence parameters on each scanner.
**Table S2**. Mean and 95% CI of bias estimate, repeatability RE, spatial heterogeneity, and reproducibility RE
**Table S3**. Corrections applied to literature values on repeatability and reproducibility to convert to definitions used in this paper.
