# Supplementary material for: Bias, Repeatability and Reproducibility of Liver T1 Mapping With Variable Flip Angles
Source: J Magn Reson Imaging. 2022 Feb 27;56(4):1042–52. doi: 10.1002/jmri.28127 (PMC9545852; doi:10.1002/jmri.28127)
Supplement: Supplementary file 1 — Table S1 Imaging protocol and optimized sequence parameters on each scanner. Table S2. Mean and 95% CI of bias estimate, repeatability RE, spatial heterogeneity, and reproducibility RE Table S3. Corrections applied to literature values on repeatability and reproducibility to convert to definitions used in this paper. [file JMRI-56-1042-s001.docx]

## Supplementary material

Table S1. Imaging protocol and optimised sequence parameters on each scanner.

| **Imaging parameters** | **S1** | **S2** | **P1** | **P2** | **G1** | **G2** |
| --- | --- | --- | --- | --- | --- | --- |
| **Field strength (T)** | **3.0** | **1.5** | **3.0** | **1.5** | **3.0** | **1.5** |
| **(i) 2D T2w coronal** | HASTE | | SSH-TSE | | SSFSE | |
| **FOV (mm×mm)** | 450×366 | | 450×366 | | 450×450 | |
| **Resolution (mm×mm)** | 1.8×1.8 | | | | | |
| **Slice thickness (mm)** | 5 | | | | | |
| **Slices** | 38 | | 38 | | 36 | |
| **TE/TR (ms)** | 87/1400 | 92/1400 | 80/1400 | 80/1400 | 89/2429 | 92/2194 |
| **Breath-holds/concatenations** | 4 | | 4 | | 5 | |
| **(ii) 2D T2w transverse** | HASTE |  | SSH-TSE | |  | |
| **FOV (mm×mm)** | 380×309 |  | 380×303 | |  | |
| **Resolution (mm×mm)** | 1.5×1.5 |  | 1.5×1.5 | |  |  |
| **Slice thickness (mm)** | 5 |  | 5 | |  |  |
| **Slices** | 35 |  | 35 | |  |  |
| **TE/TR (ms)** | 87/1400 |  | 80/1400 | |  |  |
| **Breath-holds/concatenations** | 4 |  | 3 | |  |  |
| **(iii) 2D transverse reference T1 mapping** | turboFLASH |  | MOLLI | |  | |
| **FOV (mm×mm)** | 380×309 |  | 380×309 | |  | |
| **Resolution (mm×mm)** | 1.0×1.0 |  | 2.0×2.0 | |  |  |
| **Slice thickness/Gap (mm)** | 5/20 |  | 5/20 | |  |  |
| **Slices** | 5 |  | 5 | |  |  |
| **TE/TR (ms)** | 2.75/503.73 |  | 1.05/2.24 | 1.38/3.03 |  |  |
| **Inversion times (ms)** | 100, 180, 260, 605, 685, 765, 1107, 1188, 1267, 1612, 1693, 1772, 2115, 2195, 2620, 2700, 3122, 3203, 3627, 3708, 4130, 4635, 5137, 5642, 6145, 6650, 7152, 7657 |  | 141, 350, 891, 1100, 1641, 1850, 2391, 2600, 3141, 3891, 4641 | |  |  |
| **Flip angle (°)** | 12 |  | 20 | |  |  |
| **Magnetisation preparation** | Non-sel IR T1map |  | Non-sel IR T1map | |  |  |
| **Readout** | GRE |  | bSSFP | |  |  |
| **Breath-holds or concatenations** | 5 |  | 5 | |  |  |
| **(iv) 3D coronal SPGR VFA with breath-holds** | FLASH | | T1-FFE | | MPMFA | |
| **Imaging volume (mm×mm×mm)** | 450×366×216 | | 450×366×216 | | 450×450×306 | |
| **Resolution (mm×mm×mm)** | 1.8×1.8×3 | | 1.8×1.8×3 | | 1.8×1.8×3 | |
| **TE/TR (ms)** | 1.13/3.78 | | 1.61/5.91 | | 1.05 to 1.73/3.95 to 6.04 | |
| **Parallel imaging (phase, slice)** | GRAPPA (5,1) | | SENSE (2,2) | | ASSET (3) | |
| **Flip angles (°)** | 15,25,2,5,10,20 | | | | | |
| **Time per breath-hold (or per flip angle) (s)** | 16 | | 17 | | < 18 | |
| **(v) 3D coronal SPGR VFA in free breathing** | FLASH | | T1-FFE | | MPMFA | |
| **Imaging volume (mm×mm×mm)** | 450×366×180 | | 450×375×220 | | 450×450×275 | |
| **Resolution (mm×mm×mm)** | 4.7×4.7×5 | | 4.7×4.7×5 | | 4.7×4.7×5 | |
| **TE/TR (ms)** | 0.99/3.71 | | 0.75/4.7 | | 0.68 to 0.99/3.19 to 3.97 | |
| **Parallel imaging (phase, slice)** | GRAPPA (2,2) | | SENSE (2.5,2) | | ASSET | |
| **Flip angles (°)** | 15,25,2,5,10,20 | | | | | |
| **Number of measurements per flip angle** | 36 | | 18 | | 25 | |
| **Acquisition time (mm:ss) per flip angle** | 1:01 | | 0:30 | | 1:01 | |

Table S2. Mean and 95% CI of bias estimate, repeatability RE, spatial heterogeneity, and reproducibility RE

| **Field Strength** | **Sequence** | **Vendors** | | |
| --- | --- | --- | --- | --- |
|  |  | **G** | **P** | **S** |
| **Bias estimate (%)** | | | | |
| **1.5 T** | MOLLI |  | -2.7 ± 5.5 |  |
|  | VFA BH | 31 ± 7.8 | 42 ± 12 | 0.2 ± 13 |
|  | VFA FB | 23 ± 9.0 | 26 ± 9.4 | 3.2 ± 8.3 |
| **3.0 T** | MOLLI |  | 9.2 ± 5.9 | -1.2 ± 4.5 |
|  | VFA BH | 34 ± 18 | 36 ± 19 | 44 ± 29 |
|  | VFA FB | 36 ± 13 | 34 ± 22 | 51 ± 30 |
| **Repeatability RE (%)** | | | | |
| **1.5 T** | MOLLI |  | 3.0 ± 1.5 |  |
|  | VFA BH | 12 ± 8.9 | 13 ± 7.8 | 7.6 ± 6.5 |
|  | VFA FB | 9.8 ± 5.8 | 11 ± 3.7 | 9.4 ± 6.1 |
| **3.0 T** | MOLLI |  | 2.0 ± 1.2 | 2.0 ± 1.2 |
|  | VFA BH | 6.3 ± 5.0 | 10 ± 7.9 | 16 ± 8.6 |
|  | VFA FB | 5.5 ± 2.4 | 9.7 ± 4.4 | 11 ± 6.7 |
| **Spatial Heterogeneity (%)** | | | | |
| **1.5 T** | MOLLI |  | 16 ± 1.9 |  |
|  | VFA BH | 28 ± 6.9 | 25 ± 3.2 | 18 ± 2.1 |
|  | VFA FB | 12 ± 1.6 | 15 ± 2.9 | 14 ± 1.9 |
| **3.0 T** | MOLLI |  | 10 ± 1.2 | 8.1 ± 0.9 |
|  | VFA BH | 21 ± 2.7 | 28 ± 3.1 | 28 ± 4.2 |
|  | VFA FB | 13 ± 1.6 | 25 ± 6.0 | 26 ± 5.2 |

| **Reproducibility RE (%)** | | | | | |
| --- | --- | --- | --- | --- | --- |
|  |  | **GPS** | **GP** | **PS** | **SG** |
| **1.5 T** | VFA BH | 34 ± 9.2 | 12 ± 4.7 | 44 ± 13 | 39 ± 17 |
|  | VFA FB | 24 ± 8.1 | 12 ± 5.5 | 30 ± 11 | 25 ± 12 |
| **3.0 T** | MOLLI | 14 ± 5.8 |  | 14 ± 5.8 |  |
|  | VFA BH | 22 ± 4.0 | 9.3 ± 7.3 | 24 ± 6.4 | 26 ± 8.1 |
|  | VFA FB | 29 ± 7.1 | 12 ± 8.0 | 30 ± 9.2 | 32 ± 7.3 |

Table S3. Corrections applied to literature values on repeatability and reproducibility to convert to definitions used in this paper.

|  | **Reported** | | **Corrected** | |
| --- | --- | --- | --- | --- |
| **Application area** | **Repeatability** | **Reproducibility** | **Repeatability RE** | **Reproducibility RE** |
| Breast [(8)](https://paperpile.com/c/3qSjLd/9TB8) | $wCV=100\times\frac{wSD}{mean}$  5.7% | Measured T_1_ values given in Table 3 in the paper | $CV=1.96\times wCV$  11.2% | 14% |
| Brain (R_1_) [(7)](https://paperpile.com/c/3qSjLd/Y6x8) | ${CoV}_{intra}=\frac{SD}{mean}$  5 to 10% | ${CoV}_{inter}=\frac{SD}{mean}$  5 to 10% | ${CV=1.96\times CoV}_{intra}$  10 to 20% | ${CV=1.96\times CoV}_{inter}$  10 to 20% |
| Prostate (Transitional zone) [(9)](https://paperpile.com/c/3qSjLd/zEDY) | $RC = 2.77 \times SD$   - No corrections   RC = 501  mean = 2461   - With corrections   RC = 329  mean = 1978 | $RDC = 2.77 \times SD$   - No corrections   RC = 616  mean = 2462   - With corrections   RC = 390  mean = 1978 | $CV = 1.96 \times\frac{1}{2.77}\times\frac{RC}{mean}$  12% (14% without correction) | $CV = 1.96 \times\frac{1}{2.77}\times\frac{RDC}{mean}$  14% (18% without correction) |
